# Window of opportunity trials in head and neck cancer

**DOI:** 10.20517/2394-4722.2018.100

**Published:** 2019-03-18

**Authors:** Janice L. Farlow, Andrew C. Birkeland, Paul L. Swiecicki, J. Chad Brenner, Matthew E. Spector

**Affiliations:** 1 Department of Otolaryngology - Head and Neck Surgery, University of Michigan, Ann Arbor, MI 48109, USA.; 2 Department of Otolaryngology - Head and Neck Surgery, Stanford University School of Medicine, Palo Alto, CA 94303, USA.; 3 Department of Internal Medicine, Division of Hematology and Oncology, University of Michigan, Ann Arbor, MI 48109, USA.; 4 Department of Internal Medicine, Division of Hematology and Oncology, Ann Arbor Veterans Medical Center, Ann Arbor, MI 48105, USA.

**Keywords:** Window of opportunity trial, head and neck cancer, biomarker, translational research, preoperative, oncology, trials

## Abstract

Head and neck squamous cell carcinoma (HNSCC) has a large global burden of disease and poor survival outcomes. Recent targeted therapies and immunotherapies have been explored in HNSCC, but there has been limited translation to clinical practice outside of recurrent or metastatic cases. Window of opportunity settings, where novel agents are administered between cancer diagnosis and planned definitive therapy, have begun to be employed in HNSCC. Tumor tissue biopsies are obtained at diagnosis and after the investigation treatment, along with other biospecimens and radiographic exams. Thus, this study design can characterize the safety profiles, pharmacodynamics, and initial tumor responses to novel therapies in a treatment-naïve subject. Early window studies have also identified potential biomarkers to predict sensitivity or resistance to treatments. However, these early investigations have revealed multiple challenges associated with this trial design. In this review, we discuss recent window of opportunity trials in HNSCC and how they inform design considerations for future studies.

## INTRODUCTION

**Figure 1.**
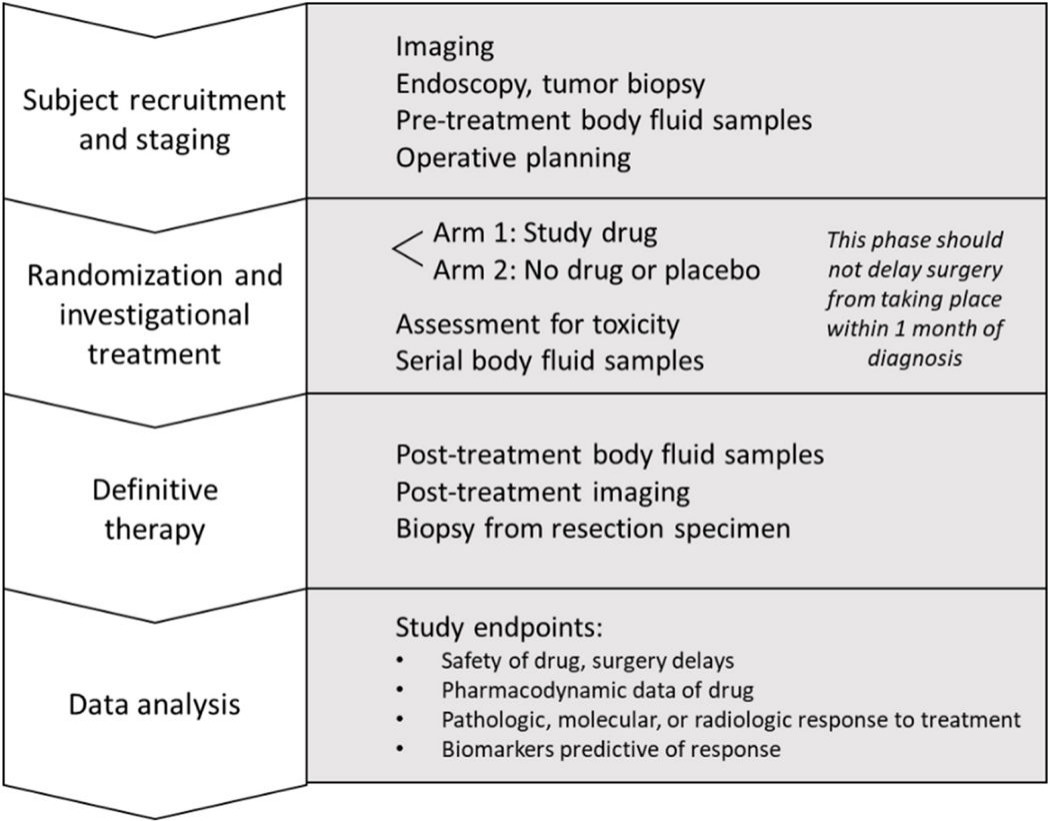
The typical format of a window of opportunity study

Head and neck squamous cell carcinoma (HNSCC) imparts significant morbidity and mortality as the sixth most common cancer in the world^[1]^. In the United States alone, over 10,000 deaths and 51,000 new cases were estimated to occur in 2018^[1]^. Nonspecific symptoms often lead to advanced stages at clinical presentation and thus poor survival outcomes, with an average 5-year survival in the United States of 65%^[2]^. Surgical extirpation is a widely employed curative approach for advanced HNSCC, but there is often a time lapse of several weeks for preoperative workup and planning. Window of opportunity or “window trials” [Figure 1] leverage this time, where normally no treatment is rendered, in order to trial novel agents without delaying standard of care therapy^[3]^ in the context of a tumor microenvironment and human pathophysiology that cannot be replicated in preclinical models. Tissue is widely available for study, given the necessity of a biopsy for initial pathologic confirmation of the diagnosis and the subsequent curative therapy. Recent genomic studies have highlighted a number of potential molecular alterations in HNSCC, which can provide valuable targets which can be studied through window trials. Additionally, immunotherapies that have shown promise in recurrent or metastatic cases can be studied in treatment-naïve subjects through this approach. Of note, neoadjuvant trials typically do not qualify as window trials, as neoadjuvant therapies are given preoperatively typically with the goal of a measurable pathologic or clinical response. Some recent neoadjuvant trials in HNSCC, however, have followed window trial methodology, in that a tumor response to therapy did not preclude or delay surgery. These trials have shed light on the safety, possible efficacy, and potential patient selection biomarkers for the therapies employed. Thus, in this article, we review select neoadjuvant and window trials in HNSCC and discuss potential future directions.

## RECENT WINDOW TRIALS OF TARGETED THERAPIES

Genomic methodologies have characterized numerous molecular alterations in HNSCC, many critical in tumor cell survival and proliferation pathways, that could form the basis for targeted therapies^[4–6]^. However, translation of these findings into clinical practice has been slow.

## ANTI-EPIDERMAL GROWTH FACTOR RECEPTOR BASED WINDOW TRIALS

Currently, the only molecularly targeted drug approved for HNSCC is cetuximab, a monoclonal antibody that targets the EGFR, an antiapoptotic transmembrane protein which is amplified or overexpressed in the vast majority of HNSCC and is correlated with reduced survival^[7]^. In a phase III trial, cetuximab has been shown to improve overall survival in HNSCC when combined with radiotherapy, although only a fraction of patients receive benefit^[8]^. Thus, identifying biomarkers that predict response to cetuximab is an ongoing area of study that potentially can be explored in a window of opportunity setting.

Schmitz *et al*.^[9]^ administered cetuximab to 33 subjects planned for curative surgery and compared radiologic tumor response via 18-fluorodeoxyglucose positron emission tomography (^18^FDG-PET) and tumor cellularity to 5 control subjects who did not receive any drug preoperatively [Table 1]. No treatment-limiting adverse events were noted, and there was a high rate of tumor response by ^18^FDG-PET (90% in cetuximab group *vs*. 0% in the control group). Tumor cellularity was correlated with ^18^FDG-PET standardized uptake values. As expected, cetuximab administration decreased pEGFR and phosphorylated extracellular signal regulated kinase expression, but neither of the biomarkers correlated with ^18^FDG-PET avidity. Cetuximab was also studied in a window of opportunity setting by Ferris *et al*.^[10]^, who noted an objective response in tumor size by computed tomography (CT) in a third of evaluable patients. Larger numbers of circulating EGFR-specific T cells^[10]^ as well as HLA class I upregulation^[11]^ were correlated with response to cetuximab.

Erlotinib is another EGFR inhibitor that has been approved in other cancers such as non-small cell lung cancer and pancreatic cancer. An uncontrolled neoadjuvant trial conducted by Thomas et al administered erlotinib in 35 subjects with advanced nonmetastatic HNSCC who were awaiting surgery^[12]^. Four subjects withdrew consent, and three subjects stopped treatment entirely due to grade 2–3 toxicities. Notably, length of treatment varied between enrolled subjects, with three subjects restarting treatment at a lower dose after grade 2–3 toxicities from the starting dose of erlotinib. Of 31 evaluable patients, decreased tumor size was seen in 9 subjects. Of multiple biomarkers studied, only the pre-erlotinib immune response score for p21^waf^, or cyclin-dependent kinase inhibitor 1, was significantly correlated with response to treatment.

Cyclooxygenase-2 (COX2) pathways are also upregulated in HNSCC, and concurrent targeting of EGFR and COX pathways has shown synergistic effects in preclinical models^[13]^. Thus, in a randomized double-blind window trial by Gross *et al*.^[13]^, 47 subjects received either erlotinib, erlotinib plus sulindac (a non-selective COX inhibitor), or placebo. One subject discontinued treatment for grade 2 anxiety, and another had their erlotinib dosage decreased for grade 2 mucositis. The primary endpoint of the Ki67 proliferation index, a cellular marker of proliferation, was only evaluable in 27 subjects. There was an ordered significant reduction of Ki67 between the erlotinib-sulindac combination *vs*. erlotinib alone, with no change in Ki67 attributable to the placebo group. No biomarkers tested appeared to mediate the decrease in Ki67, although higher pSrc expression was correlated with smaller decreases in Ki67. No clinical outcomes were evaluated.

Building from these results, Bauman *et al*.^[14]^ randomized subjects to a placebo arm or erlotinib with or without dasatinib, a small molecule inhibitor of Src family kinases. No significant treatment-altering toxicities were seen in any arm of the study. Erlotinib with or without dasatinib was correlated with a significant reduction in tumor size by response evaluation criteria in solid tumors (RECIST) measurement techniques using baseline and preoperative CT scans. Dasatinib did not appear to provide synergistic effects. No significant changes in expression levels of potential biomarker proteins was observed. Pretreatment mitogen-activated protein kinases (MAPK) and signal transducer and activator of transcription 3 expression were correlated with erlotinib sensitivity and dasatinib resistance respectively. Interestingly, the Ki67 proliferation index did not correlate with change in tumor size.

In another recent multicenter window study, Machiels *et al*.^[15]^ randomized 30 subjects to afatinib (an irreversible second generation inhibitor of the EGFR-family of receptor tyrosine kinases) or no drug prior to surgery. There were several afatinib-related adverse events, leading to discontinuation of the drug in one patient and a delay of surgery by 24 days in one subject, as well as delayed surgery with continuation of afatinib in two additional subjects. Radiologic response was seen in 16 of 23 evaluable subjects in the afatinib arm by ^18^FDG-PET and in 5 of 23 subjects by RECIST criteria. Tumor protein p53 (*TP53)* wild type allele and a hypoxia expression screen were associated with ^18^FDG-PET results but not responses by RECIST criteria.

## OTHER TARGETED WINDOW TRIALS

Uppaluri *et al*^[16]^ hypothesized that MAPK/extracellular-signal-regulated kinase (ERK) pathway could be targeted in oral cavity HNSCC. They performed a window trial^[16]^ of trametinib, an inhibitor of MAPK/ ERK kinase, that resulted in decreased tumor size by FDG avidity by PET/CT and tumor downstaging in approximately half of the 17 evaluable subjects. There were, however, 3 subjects who discontinued the study, including one who suffered a grade 4 duodenal perforation. While there was biochemical evidence of a suppressed MAPK/ERK pathway in a third of evaluable patients, no clear correlation between biochemical results and responsiveness to trametinib was drawn.

Recently, Day *et al*.^[17]^ undertook a single-armed window trial of rapamycin, an inhibitor of the mammalian target of rapamycin pathway that is dysregulated in the majority of HNSCC. Their inclusion criteria differed from the previously discussed window trials in that subjects were either planned for curative surgery (*n* = 15) or chemoradiation (*n* = 1). There was one grade 3 hypokalemia reported but no resultant delays in surgery. Decreased tumor size was seen in 14 of 16 subjects clinically and 4 of 16 patients by RECIST criteria. Ki67 was significantly decreased in all patients.

Ongoing targeted therapy window trials in HNSCC without published results include use of olaparib, a poly-ADP ribose polymerase inhibitor, and AZD6738, a serine/threonine-specific protein kinase inhibitor (NCT03022409).

## RECENT WINDOW TRIALS OF IMMUNOTHERAPIES

Studies have shown impairment of the innate and adaptive immune systems in HNSCC patients^[18]^. Immunotherapies are designed to sensitize the body’s immune system to the tumor and to counteract various strategies that tumors use to evade immunologic detection. With the recent FDA approval of nivolumab^[19]^ and pembrolizumab^[20]^ for patients with recurrent/metastatic HNSCC, there has been expansion of phase II window of opportunity trials utilizing immunomodulating drugs [Table 2]. In 2005, Timar *et al*.^[21]^ administered an interleukin-2 (IL-2) treatment to subjects with oral cavity cancer prior to surgery. Treatment consisted of peritumoral and perilymphatic injections 5 times per week over 3 weeks, along with a preceding intravenous cyclophosphamide administration and oral indomethacin and zinc sulfate medications. Matched historical pathologic specimens were used as controls. No treatment related adverse events were reported. Partial or complete response as judged by histopathologic examination or tumor dimensions on magnetic resonance imaging (MRI) were observed in 8 of 19 subjects treated with IL-2. Additionally, increased CD4+:CD8+ ratios were observed in treated subjects, although a statistically significant ratio increase between responders and non-responders was observed only in the tumor stroma. In a later study, Wolf *et al*.^[22]^ utilized subcutaneous injections of IRX-2, a biologic composed of a mixture of purified cytokines, along with cyclophosphamide, indomethacin, and zinc in a cohort of 27 patients with HNSCC. There were no significant adverse events related to treatment noted. Of 23 evaluable subjects, 4 had an objective decrease in tumor size, although this did not constitute a true partial response by RECIST criteria. Increased lymphocytic infiltration into tumors was associated with increased response and overall survival^[22,23]^.

More recently, Uppaluri *et al*.^[24]^ presented preliminary results from an ongoing single-armed trial of advanced HPV negative HNSCC subjects who received neoadjuvant pembrolizumab (an anti-PD-1 antibody) that was continued post-operatively. No serious drug-related adverse events were reported. Significantly decreased high-risk pathologic features, pathologic treatment response, and clinical-to-pathologic downstaging was observed among the 21 subjects. Baseline high tumor expression of the programmed cell death protein ligand (PD-L1) was correlated with pathologic treatment effect. Ferris *et al*.^[25]^ presented interim results of a window trial of nivolumab (another anti-PD-1 antibody) for HNSCC. In half of the 23 evaluable subjects, tumor dimensions were reduced after treatment. As part of another ongoing neoadjuvant trial, Colevas *et al*.^[26]^ are administering anti-PD-1 antibody prior to planned curative surgery or radiation in HNSCC. They presented results from a single subject where their novel nuclear medicine imaging test correlated with tissue markers of immunologic activity.

A novel antibody MEDI6469, an OX40 (CD134) agonist, was also studied in a window of opportunity setting by Bell *et al*.^[27]^. No significant adverse events were reported, and immunologic response was detected in 4 of 17 subjects. There was a significant difference between responders and non-responders in genes associated with major histocompatibility complex (MHC I)-mediated antigen processing.

Shayan *et al*.^[28]^ combined motolimod, a small molecule agonist of the toll-like receptor 8, along with cetuximab in 14 patients planned for curative surgery. One subject withdrew from the study due to an unspecified cetuximab toxicity. Study results showed that the expected increase in suppressive co-signaling molecule expression induced by cetuximab monotherapy was counteracted by the addition of motolimod, resulting in increased circulating EGFR-specific T cells and greater tumor infiltration of leukocytes.

## OTHER WINDOW TRIALS

While not a classic targeted therapy or immunotherapy, metformin, an anti-hyperglycemic, has been shown to be associated with improved outcomes in HNSCC^[29]^. It is postulated that metformin’s metabolic effects through inhibition of mitochondrial oxidative phosphorylation may be proapoptotic in HNSCC. Curry *et al*.^[30]^ executed a single-armed window trial of metformin among 39 subjects with HNSCC of the oral cavity or larynx, each who took between 9–24 days of the drug without significant side effects. Markers of increased apoptosis and altered stromal metabolism were identified in the 33 evaluable subjects.

The results of several additional targeted therapy and immunotherapy trials have yet to be published. Those listed in ClinicalTrials.gov are briefly reviewed in Table 3.

## CONSIDERATIONS IN DESIGNING FUTURE WINDOW TRIALS

Window trials offer the opportunity to study the safety, mechanism, and efficacy of novel agents in treatment-naïve HNSCC. The studies outlined here have demonstrated the overall safety of each agent studied, with limited numbers of treatment-related adverse events and no clear post-operative complications attributable to the investigational drug. They have also confirmed the intended knockdown of upregulated pathways in HNSCC with targeted therapies and have shed light on the immunomodulatory mechanisms behind newer immunotherapies. Promising preliminary data reveal clinical, radiologic, and pathologic responses in some treated subjects along with possible biomarkers predictive of sensitivity or resistance to the studied agents, although work remains to duplicate and understand these results.

By definition, window trials occur in a short timeframe, which requires careful coordination to obtain the desired imaging studies, tumor tissue, and serial biologic samples. As the authors of a recent study discuss^[15,31]^, this can be difficult in a patient population that often has socioeconomic and adherence challenges with an already complicated diagnosis and treatment strategy to discuss. For this reason and because patients may be hesitant to take an investigational drug that should not be marketed to improve clinical outcomes in the research setting, accrual can take longer than expected. Accrual goals were not uniformly available for the studies included in this review, and many unpublished planned window trials may have failed due to poor accrual. Narrowing subject selection to specific tumor sites (i.e., oral cavity, oropharynx, larynx, or hypopharynx) or immunogenomic profiles may further elongate recruitment timelines.

Pre- and post-treatment tissue is readily available by nature of the window of opportunity design, but the timing, selection, processing, and analysis protocols for tumor tissue and other desired body fluid samples must be considered. Tumor heterogeneity is a well-known phenomenon, and immunogenomic profiles can vary across both space and time. Pharmacokinetics of the drug under study should also be factored into the timing of obtaining biologic samples. Unlike in breast cancer where Ki67 is commonly employed, HNSCC studies have not coalesced on particular biomarkers, nor do standardized protocols for obtaining biomarker data or evaluating their clinical impact exist as of yet^[14,15]^. Several window trials discussed here were not randomized or did not use data from control subjects, which has been known to complicate pharmacodynamic and predictive biomarker assessment^[13]^. Studies presented herein have also collected serum samples, but analytes from other body fluid samples that could serve as future “liquid biopsies^[32]^” have yet to be characterized in window trials.

Given that there may be physical reduction in tumor size from the drug under study, it is important to confirm with subjects that surgery is still required as part of the study even if the tumor shrinks or disappears radiographically, and surgical margins should be based on pre-treatment tumor dimensions^[31]^. Similar to biologic samples, the type, timing, protocols, and quality thresholds for radiologic tests must be carefully planned, particularly if imaging from multiple institutions are used. The window trials presented here used a variety of exams, including CT, MRI with different protocols, ^18^FDG-PET, and investigational PET technologies. Additionally, criteria for assessing radiologic response included those from RECIST, modified RECIST^[14]^, EORTC (European Organization for Research and Treatment of Cancer)^[15]^, the World Health Organization^[16]^, and others. Researchers should also be aware that pseudoprogression during immunotherapy, or an initial tumor flare due to inflammatory processes provoked by the drug, may complicate image interpretation during the short timeframe of a window study^[33,34]^. This should not be confused with hyperprogression, a phenomenon of tumor growth during immunotherapy treatment experienced by a small minority of patients, which may delay curative surgery^[34,35]^. Limited data are available on the optimal timing of surgery, but it is suggested that HNSCC resection should take place within a month of diagnosis^[36,37]^. Treatment-related adverse events beyond hyperprogression may delay curative surgery, so investigational drugs selected for window trials should have well-characterized safety data and a tolerable safety margin. Trial stopping points based on safety events should be well-defined and monitored by an independent committee.

Finally, it is important to note that window trials cannot assess the long-term response, acquired resistance mechanisms, or safety profile for the studied treatment. Complementary study designs should be utilized to contextualize window trial results. For instance, a single window trial may provide compelling preliminary data for a full confirmatory neoadjuvant trial. The I-SPY2 TRIAL (Investigation of Serial Studies to Predict Your Therapeutic Response with Imaging and Molecular Analysis)^[38]^ utilizes this approach in breast cancer, with the added benefit of conducting studies on multiple agents in parallel. This technique could be applied in HNSCC, although the window trial approach is likely most effective for treatment-naïve and healthier patient populations. Biomolecular insights gained from window trials, on the other hand, could inform pathophysiology in multiple patient populations, as well as subject/agent selection for all types of clinical trial designs.

## CONCLUSION

Window of opportunity studies are challenging to design and execute. Despite this, early window trials have explored the safety, pharmacodynamics, short-term efficacy, and predictive biomarkers for novel targeted therapies and immunotherapies. Window trials are a promising study design complementary to traditional clinical trials to advance understanding and treatment of HNSCC.

## Figures and Tables

**Table 1.** Window trials of targeted therapies in head and neck squamous cell carcinoma

Ref.	Agent(s)	*n* ^*^	Inclusion criteria	Duration	Outcome(s)	Biomarkers	Toxicity/surgery delays
Day *et al*.^[17]^	Rapamycin	16 (21^‡^)	Stage II-IV OC/OP	21 days	Tumor size (clinically, CT/RECIST)	NS	
Machiels *et al.*^[15]^	1. Afatinib 2. No drug	25 5 (30)	Stage II-IV OC/OP	14 days	^18^FDG-PET response (EORTC, RECIST); DCEMRI; DW-MRI	TP53 genotype, hypoxia screen	G3 diarrhea, renal failure (*n* = 1), surgery delay (*n* = 3)
Bauman *et al.*^[14]^	1. Erlotinib 2. Desatinib 3. Combination 4. Placebo	14 15 14 15 (56)	Stage I-IV OC/OP/L	21 days	Tumor size (CT)	pMAPK, pSTAT3	None
Uppaluri *et al*.^[16]^	Trametinib	20 (20)	Stage II-IV OC	7–16 days	Tumor size, SUV (PET/CT; WHO); tumor stage	NR	G4 duodenal perforation (*n* = 1), G2 nausea (*n* = 1); 3 patients stopped treatment
Ferris *et al.*^[10]^	Cetuximab	40 (40)	Stage III-IV OC/OP/ L/HP	21–28 days	Tumor size (CT); progression free survival	EGFR-specific T cell counts	NR
Gross *et al.*^[13]^	1. Erlotinib 2. Erlotinib + Sulindac 3. Placebo	19 16 12 (39^†^)	Stage II-IVA OC/ OP/L/HP	7–14 days	Ki67 proliferation index	pSrc	G2 anxiety (*n* = 1 stopped treatment), G2 mucositis (*n* = 1 decreased dosage)
Schmitz *et al.*^[9]^	1. Cetuximab 2. No drug	33 5	T1-T4 OC/OP/L/HP	21 days	^18^FDG-PET response (SUV); tumor size (CT/ MRI); tumor cellularity	NS	None
Thomas *et al.*^[12]^	Erlotinib	35	T2-T4 OC/OP/L/HP	18–29 days	Tumor size (CT)	p21^waf^	G3 pruritis and G2 rash (*n* = 6; *n* = 3 stopped treatment)

Studies listed by date published.

‡ *n* = 21 and *n* = 37 listed as accrual number and actual enrollment on ClinicalTrials.gov, with *n* = 16 included in the published manuscript

† accrual number modified based on discontinuation of parent study

* sample sizes listed include actual number of subjects, with the amount necessary for full accrual in parentheses if published.

Biomarkers listed in the table include biologic characteristics statistically associated with sensitivity or resistance to the tested therapy. Toxicities only include those attributed to or possibly attributed to the drug being studied that are grade (G) 3 or higher or caused treatment dosage reduction or discontinuation. Ref.: reference; HNSCC: head and neck squamous cell carcinoma; OC: oral cavity; OP: oropharynx; P: pharynx; HP: hypopharynx; L: larynx; CT: computed tomography; ^18^FDG-PET: 18-fluorodeoxyglucose-positron emission tomography; SUV: standardized uptake value; DCE-MRI: dynamic contrast enhanced magnetic resonance imaging; DW-MRI: diffusion-weighted MRI; RECIST: response evaluation criteria in solid tumors; EORTC: European Organization for Research and Treatment of Cancer; WHO: World Health Organization; NS: not significant; NR: not reported

**Table 2.** Window trials of immunotherapies in head and neck squamous cell carcinoma

Ref.	Agent(s)	*n* ^*^		Inclusion criteria	Duration	Outcome(s)	Biomarkers	Toxicity/ surgery delays
Bell *et al*.^[27]^	Anti-OX40 (MEDI6469)	17 (55^‡^)		Stage III-IV	5–6 days	TIL counts and expression profiles	MHC I genes	None
Colevas *et al*.^[26]^	Anti-PD-1 Ab	NR^‡^		NR	NR	TIL counts and expression profiles	Novel PET imaging	NR
Shayan *et al*.^[28]^	Motolimod + cetuximab	14		Stage III-IV OC/ OP/L/HP	15–22 days	TIL counts, circulating leukocytes, immune effector cell biomarkers		Unspecified cetuximab toxicity (*n* = 1, withdrew from study)
Ferris *et al*.^[25]^	Nivolumab	29^‡^		T1+N1+ OC/P/L	15 days	Tumor size (CT) Pathologic response Tumor PD-L1 expression Immune correlates	NR	Grade 3–4 adverse events (*n* = 4)
Uppaluri *et al*.^[24]^	Pembrolizumab	21^‡^		Stage III-IV HPV negative	NR	High-risk pathologic features Pathologic treatment response Tumor staging	Baseline PD-L1 expression	None
Berinstein *et al*.^[23]^ Wolf *et al*.^[22]^	IRX-2	27	Stage II-IV OC/ OP/L/HP		21 days	Tumor size (CT/MRI; RECIST), TIL counts		Postoperative wound infection (*n* = 1)
Timar *et al*.^[21]^	1. IL-2 2. Historical pathologic controls	19 20		T2–3 OC	21 days	Pathologic analysis, Tumor dimensions (MRI)	CD4:CD8 ratio	None

Studies listed by date published.

‡ Active study on ClinicalTrials.gov

* sample sizes listed include actual number of subjects, with the amount necessary for full accrual in parentheses if published.

Biomarkers listed in the table include biologic characteristics statistically associated with sensitivity or resistance to the tested therapy. Toxicities only include those attributed to or possibly attributed to the drug being studied that are grade (G) 3 or higher or caused treatment dosage reduction or discontinuation. Ref.: reference; HNSCC: head and neck squamous cell carcinoma; OC: oral cavity; OP: oropharynx; P: pharynx; HP: hypopharynx; L: larynx; TIL: tumor infiltrating leukocyte; CT: computed tomography; PET: positron emission tomography; MRI: magnetic resonance imaging; RECIST: response evaluation criteria in solid tumors; NR: not reported

**Table 3.** Ongoing window trials in head and neck squamous cell carcinoma

Type	Principal investigator/institution	Agent(s)	Inclusion criteria^*^	NCT
Targeted therapy	Duvurri/University of Pittsburgh	AZD6738, olaparib	Newly diagnosed, treatment naive Planned surgery/biopsy + adjuvant RT and/or chemo	03022409
Immunotherapy	Wolf/University of Michigan	IRX-2	Stage II-IVA OCSCC Treatment naive KPS ≥ 70% Adequate hematologic, hepatic, and renal function	02609386
Immunotherapy	Worden/University of Michigan	Pembrolizumab	Any T stage with ≥ N2 disease T4 disease, any N stage T3 OCSCC, any N stage Clinical evidence of ECE ECOG 0–1	02641093
Immunotherapy	Neskey/Medical University of South Carolina	Nivolumab	Newly diagnosed, treatment naive, T2-T4, M0 OCSCC; or Recurrent/persistent locoregional T2-T4 OCSCC initially treated with surgery alone, ECOG 0–1	03021993
Immunotherapy	Schoenfeld/Dana- Farber Cancer Institute	Nivolumab ± Ipilimumab	≥ T2 ± ≥ N1 surgically resectable OCSCC ECOG 0–1 Adequate hematologic, hepatic, and renal function	02919683
Immunotherapy	Porosnicu/Wake Forest	Durvalumab	Surgically resectable OCSCC/OPSCC No prior immunotherapy or RT ECOG 0–1 Adequate hematologic, hepatic, and renal function	02827838
Immunotherapy	Curry/Thomas Jefferson	Durvalumab ± Metformin	Surgically resectable HNSCC ECOG 0–1 Body weight > 30 kg Adequate hematologic, hepatic, and renal function	03618654
Targeted therapy/ immunotherapy	Ferris/University of Pittsburgh	Motolimod and Cetuximab ± Nivolumab	Treatment naive Stage II-IVA HNSCC Planned surgical resection ECOG 0–1 Adequate hematologic, hepatic, and renal function	02124850

* Inclusion criteria abbreviated. See ClinicalTrials.gov for full inclusion and exclusion criteria, as well as primary endpoints for each trial.

HNSCC: head and neck squamous cell carcinoma; NCT: ClinicalTrials.gov identifier; OCSCC: oral cavity SCC; OPSCC: oropharyngeal SCC; P: pharynx; HP: hypopharynx; L: larynx; KPS: Karnofsky performance status; ECOG: Eastern Cooperative Oncology Group Performance Scale; RT: radiotherapy; ECE: extracapsular extension

## References

[1] Siegel RL, Miller KD, Jemal A. Cancer statistics, 2018. CA Cancer J Clin 2018;68:7–30. 29313949 10.3322/caac.21442

[2] Pulte D, Brenner H. Changes in survival in head and neck cancers in the late 20th and early 21st century: a period analysis. Oncologist 2010;15:994–1001. 20798198 10.1634/theoncologist.2009-0289 PMC3228039

[3] Kalinsky K, Hershman DL. Cracking open window of opportunity trials. J Clin Oncol 2012;30:2573–5. 22565006 10.1200/JCO.2012.42.3293

[4] Cancer Genome Atlas N. Comprehensive genomic characterization of head and neck squamous cell carcinomas. Nature 2015;517:576–82. 25631445 10.1038/nature14129 PMC4311405

[5] Hoesli RC, Ludwig ML, Michmerhuizen NL, Rosko AJ, Spector ME, Genomic sequencing and precision medicine in head and neck cancers. Eur J Surg Oncol 2017;43:884–92. 28034498 10.1016/j.ejso.2016.12.002 PMC5393934

[6] Stransky N, Egloff AM, Tward AD, Kostic AD, Cibulskis K, The mutational landscape of head and neck squamous cell carcinoma. Science 2011;333:1157–60. 21798893 10.1126/science.1208130 PMC3415217

[7] Chung CH, Ely K, McGavran L, Varella-Garcia M, Parker J, Increased epidermal growth factor receptor gene copy number is associated with poor prognosis in head and neck squamous cell carcinomas. J Clin Oncol 2006;24:4170–6. 16943533 10.1200/JCO.2006.07.2587

[8] Bonner JA, Harari PM, Giralt J, Azarnia N, Shin DM, Radiotherapy plus cetuximab for squamous-cell carcinoma of the head and neck. N Engl J Med 2006;354:567–78. 16467544 10.1056/NEJMoa053422

[9] Schmitz S, Hamoir M, Reychler H, Magremanne M, Weynand B, Tumour response and safety of cetuximab in a window pre-operative study in patients with squamous cell carcinoma of the head and neck. Ann Oncol 2013;24:2261–6. 23704200 10.1093/annonc/mdt180

[10] Ferris RL, Kim S, Trivedi S, Srivastava RM, Concha-Benavente F, Correlation of anti-tumor adaptive immunity with clinical response in a phase II “window” trial of neoadjuvant cetuximab in ptaients with resectable stage III-IV head and neck squamous carcinoma (HNSCC). J Clin Oncol 2016;34:6060.

[11] Srivastava RM, Trivedi S, Concha-Benavente F, Hyun-Bae J, Wang L, STAT1-Induced HLA Class I Upregulation Enhances Immunogenicity and Clinical Response to Anti-EGFR mAb Cetuximab Therapy in HNC Patients. Cancer Immunol Res 2015;3:936–45. 25972070 10.1158/2326-6066.CIR-15-0053 PMC4526378

[12] Thomas F, Rochaix P, Benlyazid A, Sarini J, Rives M, Pilot study of neoadjuvant treatment with erlotinib in nonmetastatic head and neck squamous cell carcinoma. Clin Cancer Res 2007;13:7086–92. 18056187 10.1158/1078-0432.CCR-07-1370

[13] Gross ND, Bauman JE, Gooding WE, Denq W, Thomas SM, Erlotinib, erlotinib-sulindac versus placebo: a randomized, double-blind, placebo-controlled window trial in operable head and neck cancer. Clin Cancer Res 2014;20:3289–98. 24727329 10.1158/1078-0432.CCR-13-3360 PMC4104657

[14] Bauman JE, Duvvuri U, Gooding WE, Rath TJ, Gross ND, Randomized, placebo-controlled window trial of EGFR, Src, or combined blockade in head and neck cancer. JCI Insight 2017;2:e90449. 28352657 10.1172/jci.insight.90449 PMC5358497

[15] Machiels JP, Bossi P, Menis J, Lia M, Fortpied C, Activity and safety of afatinib in a window preoperative EORTC study in patients with squamous cell carcinoma of the head and neck (SCCHN). Ann Oncol 2018;29:985–91. 29346507 10.1093/annonc/mdy013

[16] Uppaluri R, Winkler AE, Lin T, Law JH, Haughey BH, Biomarker and Tumor Responses of Oral Cavity Squamous Cell Carcinoma to Trametinib: A Phase II Neoadjuvant Window-of-Opportunity Clinical Trial. Clin Cancer Res 2017;23:2186–94. 28151720 10.1158/1078-0432.CCR-16-1469 PMC5509449

[17] Day TA, Shirai K, O’Brien PE, Matheus MG, Godwin KB, Inhibition of mTOR Signaling and Clinical Activity of Rapamycin in Head and Neck Cancer in a Window of Opportunity Trial. Clin Cancer Res 2019;25:1156–64. 30420444 10.1158/1078-0432.CCR-18-2024 PMC6377824

[18] Ferris RL. Immunology and Immunotherapy of Head and Neck Cancer. J Clin Oncol 2015;33:3293–304. 26351330 10.1200/JCO.2015.61.1509 PMC4586169

[19] Ferris RL, Blumenschein G Jr., Fayette J, Guigay J, Colevas AD, Nivolumab for Recurrent Squamous-Cell Carcinoma of the Head and Neck. N Engl J Med 2016;375:1856–67. 27718784 10.1056/NEJMoa1602252 PMC5564292

[20] Cohen EEW, Soulieres D, Le Tourneau C, Dinis J, Licitra L, Pembrolizumab versus methotrexate, docetaxel, or cetuximab for recurrent or metastatic head-and-neck squamous cell carcinoma (KEYNOTE-040): a randomised, open-label, phase 3 study. Lancet 2019;393:156–67. 30509740 10.1016/S0140-6736(18)31999-8

[21] Timar J, Ladanyi A, Forster-Horvath C, Lukits J, Dome B, Neoadjuvant immunotherapy of oral squamous cell carcinoma modulates intratumoral CD4/CD8 ratio and tumor microenvironment: a multicenter phase II clinical trial. J Clin Oncol 2005;23:3421–32. 15908653 10.1200/JCO.2005.06.005

[22] Wolf GT, Fee WE Jr., Dolan RW, Moyer JS, Kaplan MJ, Novel neoadjuvant immunotherapy regimen safety and survival in head and neck squamous cell cancer. Head Neck 2011;33:1666–74. 21284052 10.1002/hed.21660 PMC4062188

[23] Berinstein NL, McNamara M, Nguyen A, Egan J, Wolf GT. Increased immune infiltration and chemokine receptor expression in head and neck epithelial tumors after neoadjuvant immunotherapy with the IRX-2 regimen. Oncoimmunology 2018;7:e1423173. 29721379 10.1080/2162402X.2017.1423173 PMC5927542

[24] Uppaluri R, Zolkind P, Lin T, Nussenbaum B, Jackson RS, Neoadjuvant pembrolizumab in surgically resectable, locally advanced HPV negative head and neck squamous cell carcinoma (HNSCC). J Clin Oncol 2017;35:6012.

[25] Ferris RL, Goncalves A, Baxi SS, Martens UM, Gauthier H, LBA46 - an open-label, multicohort, phase 1/2 study in patients with virus-associated cancers (CheckMate 358): safety and efficacy of neoadjuvant nivolumab in squamous cell carcinoma of the head and neck. Ann Oncol 2017;28:v605–v49.

[26] Colevas AD, Bedi N, Chang S, Nieves UYM, Chatterjee S, A study to evaluate immunological response to PD-1 inhibition in squamous cell carcinoma of the head and neck (SCCHN) using novel PET imaging with [18F]F-AraG. J Clin Oncol 2018;36:6050.

[27] Bell RB, Duhen R, Leidner RS, Curti BD, Ballesteros-Merino C, Neoadjuvant anti-OX40 (MEDI6469) prior to surgery in head and neck squamous cell carcinoma. J Clin ONcol 2018;36:6011.

[28] Shayan G, Kansy BA, Gibson SP, Srivastava RM, Bryan JK, Phase Ib Study of Immune Biomarker Modulation with Neoadjuvant Cetuximab and TLR8 Stimulation in Head and Neck Cancer to Overcome Suppressive Myeloid Signals. Clin Cancer Res 2018;24:62–72. 29061643 10.1158/1078-0432.CCR-17-0357 PMC5754237

[29] Rego DF, Pavan LM, Elias ST, De Luca Canto G, Guerra EN. Effects of metformin on head and neck cancer: a systematic review. Oral Oncol 2015;51:416–22. 25636350 10.1016/j.oraloncology.2015.01.007

[30] Curry J, Johnson J, Tassone P, Vidal MD, Menezes DW, Metformin effects on head and neck squamous carcinoma microenvironment: Window of opportunity trial. Laryngoscope 2017;127:1808–15. 28185288 10.1002/lary.26489 PMC5515672

[31] Schmitz S, Caballero C, Locati LD. Perspectives on window of opportunity trials in head and neck cancer: lessons from the EORTC 90111–24111-NOCI-HNCG study. Eur J Cancer 2018;104:219–23. 30301582 10.1016/j.ejca.2018.07.315

[32] Spector ME, Farlow JL, Haring CT, Brenner JC, Birkeland AC. The potential for liquid biopsies in head and neck cancer. Discov Med 2018;25:251–7. 29906408 PMC6125134

[33] Baxi SS, Dunn LA, Burtness BA. Amidst the excitement: A cautionary tale of immunotherapy, pseudoprogression and head and neck squamous cell carcinoma. Oral Oncol 2016;62:147–8. 27776933 10.1016/j.oraloncology.2016.10.007

[34] Hanna GJ, Adkins DR, Zolkind P, Uppaluri R. Rationale for neoadjuvant immunotherapy in head and neck squamous cell carcinoma. Oral Oncol 2017;73:65–9. 28939078 10.1016/j.oraloncology.2017.08.008

[35] Saada-Bouzid E, Defaucheux C, Karabajakian A, Coloma VP, Servois V, Hyperprogression during anti-PD-1/PD-L1 therapy in patients with recurrent and/or metastatic head and neck squamous cell carcinoma. Ann Oncol 2017;28:1605–11. 28419181 10.1093/annonc/mdx178

[36] Schmitz S, Duhoux F, Machiels JP. Window of opportunity studies: Do they fulfil our expectations? Cancer Treat Rev 2016;43:50–7. 26827692 10.1016/j.ctrv.2015.12.005

[37] Primdahl H, Nielsen AL, Larsen S, Andersen E, Ipsen M, Changes from 1992 to 2002 in the pretreatment delay for patients with squamous cell carcinoma of larynx or pharynx: a Danish nationwide survey from DAHANCA. Acta Oncol 2006;45:156–61. 16546860 10.1080/02841860500423948

[38] Esserman LJ, Woodcock J. Accelerating identification and regulatory approval of investigational cancer drugs. JAMA 2011;306:2608–9. 22187281 10.1001/jama.2011.1837

